# Four “teachable moments” for “planetary health” or how to integrate sustainable healthcare topics into clinical courses in family medicine for undergraduate medical students

**DOI:** 10.3205/zma001781

**Published:** 2025-11-17

**Authors:** Telemachos Hatziisaak, Olivier Pasche, Andreas Plate, Baptiste Pedrazzini, Luca Gabutti, Arabelle Rieder, SAFMED Teaching Group (Swiss Academy for Family Medicine)

**Affiliations:** 1 Universität St. Gallen (HSG), School of Medicine, St. Gallen, Switzerland; 2 University of Fribourg (UNIFR), Institute of Family Medicine, Fribourg, Switzerland; 3 University Hospital Zurich (USZ) and University Zurich (UZH), Institute for Family Medicine, Zurich, Switzerland; 4 University of Lausanne (UNIL), Department of Family Medicine, University Center for General Medicine and Public Health (Unisanté), Lausanne, Switzerland; 5 University of Italian Switzerland (USI), Institute of Family Medicine, Lugano, Switzerland; 6 University of Geneva (UNIGE), University Institute of Family Medicine and Child Health, Geneva, Switzerland

**Keywords:** planetary health, sustainable healthcare, climatechange, family medicine, undergraduate medical education

## Abstract

This article discusses the integration of planetary health and sustainable healthcare into family medicine education, focusing on undergraduate medical training. While environmental impacts on health have been recognized since ancient times, the urgency of addressing climate change in healthcare has escalated, especially as the healthcare sector contributes significantly to greenhouse gas emissions. The article highlights practical ways for medical educators to incorporate sustainable healthcare practices in family medicine settings. Through four “teachable moments”, it demonstrates how medical students can engage in sustainability efforts, from reducing practice carbon footprints to minimizing unnecessary tests and treatments. The challenges of incorporating planetary health into an already crowded medical curriculum are acknowledged, and the authors propose an integrative approach, leveraging family practices’ flexibility. They emphasize the importance of involving students in sustainability efforts, fostering bidirectional learning that benefits both the teaching practice and the students’ future careers.

## Abstract (de)

Der vorliegende Artikel befasst sich mit der Integration der Themen „Planetary Health“ und „nachhaltige Gesundheitsversorgung“ in die hausärztliche Ausbildung im Rahmen des Medizinstudiums. Während die Auswirkungen von Umweltfaktoren auf die Gesundheit schon seit der Antike bekannt sind, hat die Dringlichkeit, sich mit dem Klimawandel auseinanderzusetzen auch im Gesundheitssystem zugenommen, da dieses erheblich zu den Treibhausgasemissionen beiträgt. Der Artikel zeigt praktische Möglichkeiten auf, wie in der medizinischen Ausbildung nachhaltiges Handeln in die hausärztliche Lehre einbezogen werden kann. Anhand von vier „Teachable Moments“ wird aufgezeigt, wie Medizinstudierende sich für Nachhaltigkeit engagieren können, von der Reduktion des CO_2_-Fußabdrucks der Praxis bis zum Weglassen unnötiger Untersuchungen und Behandlungen. Die Autoren sind sich der Herausforderungen bewusst, die der Einbezug des Themas „Planetary Health“ in ein bereits überfülltes medizinisches Curriculum mit sich bringt, und schlagen einen integrativen Ansatz vor, der die Flexibilität der Hausarztpraxis nutzt. Sie betonen die Wichtigkeit, Studierende in die Bemühungen um nachhaltige Medizin einzubeziehen, wodurch ein bidirektionales Lernen entsteht, von dem sowohl die Lehrpraxis als auch die Studierenden für ihre spätere Tätigkeit profitieren.

## Introduction

Planetary health is on everyone’s lips. The Swiss Medical Association (FMH) is also dealing with it [1]. But planetary health is nothing new. Concern about the environment as a risk factor for health has preoccupied mankind in general and the medical profession in particular since Hippocrates [2]. And since industrialization at the latest, everyone has been aware that manmade environmental pollution makes living beings ill – think of smog, heavy metals in the soil, microplastics in water or radiation disasters, to name just a few examples. In Europe, awareness of the issue of environmental protection as a source for health promotion has been raised among the population and the medical profession since the 1970s in the course of the emergence of the ecological movement ([https://environmentalhistory.org/20th-century/seventies-1970-79/], retrieved 8^th^ January 2025).

Over the last few years awareness in the scientific community about the magnitude of the phenomenon of climate change and the risks hanging over humanity has changed dramatically, making it a real emergency. In fact, the Intergovernmental Panel on Climate Change (IPCC) report published in 2023 shows that the objectives of the Paris agreements signed in 2015 cannot be met. Even more worryingly, one scenario that seems the most realistic today predicts an increase in average temperatures of +4°C to +5°C by 2100, which would mean the transformation of terrestrial and aquatic environments of a large part of the globe into areas incompatible with life [3]. Given the significant contribution made by the healthcare system to the total production of greenhouse gases (GHG) in European countries (about 5% of the total mass), it is essential that savings measures be taken in this sector.

Ambulatory care contributes to 18% of healthcare emissions [4]. In a study conducted by Nicolet et al, analyzing 10 family medicine practices in the Swiss Canton Vaud, the average annual GHG production was found to be 30.5 CO2e tons, with more than half (55.5%) attributed to mobility (33.2% from patients, 12.5% from staff), while the second source was the heating system (29.8%) [5].

Regarding pharmaceutical products, the major pollutants in ambulatory care are pressurized metered-dose inhalers (MDIs), due to the hydrofluoroalkane propellants released into the atmosphere during use [6]. Other drugs contribute approximately to 10%-20% of healthcare emissions [7], primarily due to production, packaging, and transportation systems specific to each pharmaceutical company but not to a single medication. Another way in which medications contribute to global pollution is through improper disposal: in this case, the main culprits are antibiotics [8], which contaminate water systems once released into the environment.

It will be necessary to explore ways of significantly reducing the consumption of medication, given the ecological burden they create within the healthcare system [9].

In medical practices sustainable healthcare therefore is an emerging topic. In an environment characterized by a throwaway society and a growingly demanding stance from the patients’ side, fueled by ubiquitous media consumption, sticking to and promoting sustainable healthcare issues is a veritable challenge.

Unfortunately, economic incentives to act in that sense are limited. There is of course a wealth of possibilities that falls into the area of personal willingness to behave sustainably towards the environment. This starts with the measured use of crepe paper on the examination table and ends with the proper disposal of potentially infectious material, so as not to harm practice staff.

The FMH and the Swiss Institute for Medical Education (SIME) are prepared to play an active part in raising awareness of the health impacts of climate change across all generations in the medical profession, and to take relevant action and support measures. This requires awareness of these processes at all levels, from policymaking, associations and organisations, and universities or training institutions, to surgeries and hospitals [10].

Given the importance of what is at stake, our family medicine community should play its part and the issue should be addressed in the training of future doctors. But where should we start? And how can the topic of ecological behavior be integrated into undergraduate medical education, given the fact that the SIME is not responsible for undergraduate training in human medicine?

As clinical teachers in family medicine, we have multiple encounters with medical students and trainees during clinical placements in our practices. This provides unique opportunities to exchange knowledge and skills with the new generation of doctors. Thus, in the following paragraphs, we invite you to follow Sabine, a 4^th^ year medical student who has been assigned to your practice for 10 half-days. You wish to implement recent guidelines on sustainable healthcare that you have read about in a medical journal. You also realize that Sabine has probably more knowledge about environmental issues than you through her own research and medical school training. How can this new subject become a mutual learning opportunity?

## First “teachable moment” for “planetary health”: The FMH toolkit

**Figure 1:**
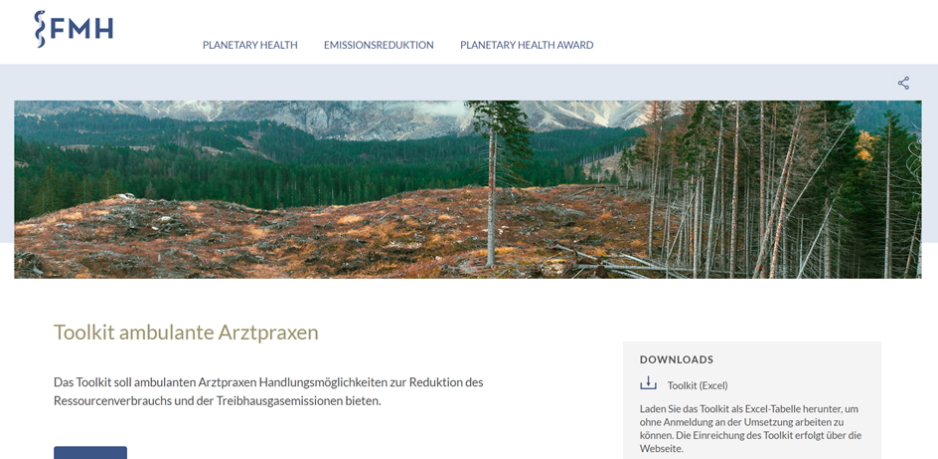
Website of the Swiss Medical Association (FMH) with the Planetary Health Toolkit: [https://toolkit.fmh.ch/] (retrieved 8^th^ January 2025)

On Sabine’s first half-day you show her around the practice and introduce her to your other colleagues and to your medical-assistants at the reception. After you have made her feel welcome and discussed her learning objectives before she starts consultations with you, you mention a new project to reduce the practice’s carbon footprint. This is based on recommendations published by the FMH called the “FMH toolkit” ([https://toolkit.fmh.ch/], retrieved 8^th^ January 2025) (see figure 1 (Fig. 1))

The practice team has already identified ways to save electricity and warm water consumption, to avoid unnecessary use of disposable plastic and encourage patients to use public transport. For home visits the doctors even use a small electric vehicle instead of cars with combustion engines.

Sabine reads the list of changes already proposed by the practice team and shows her interest in participating in the next planned meeting to discuss the progress of the project.

During the meeting, Sabine realizes that it is difficult for the senior members of the team to apply the recommendation to avoid systematically printing out documents. The problem lies in using the yet unknown new functions of the electronic records. Sabine volunteers to look over the new procedure with members of the team who are interested and help them with the steps to send documents directly from the medical file to the patients email via a secured procedure.

If a document still needs to be printed, Sabine recommends that all white paper purchased by the practice should meet at least one of the following options: It is made either from 70% or NAPM-certified recycled paper or from pulp from FSC-certified sustainable sources.

When you discuss Sabine’s contribution to the implementation of the project to improve sustainability, your student says that she was pleased she was given a chance to participate in a small way in the project and felt encouraged to bring up the subject of planetary health in future placements.

## Second “teachable moment” for “planetary health”: 12 months/12 actions

Since January 2024, the Revue Médicale Suisse (RMS), has supported an initiative to provide evidence-based “green prescriptions” on 12 themes that are beneficial for both the individual health of patients and the environment ([https://www.revmed.ch/infos-patients/calendrier-12-mois-12-actions-pour-l-environnement], retrieved 8^th ^January 2025). Each month, a new action is proposed and presented on the RMS website and as a printed colourful poster included in the journal to be displayed in the medical practices. The posters have also been translated into German and Italian.

**Figure 2:**
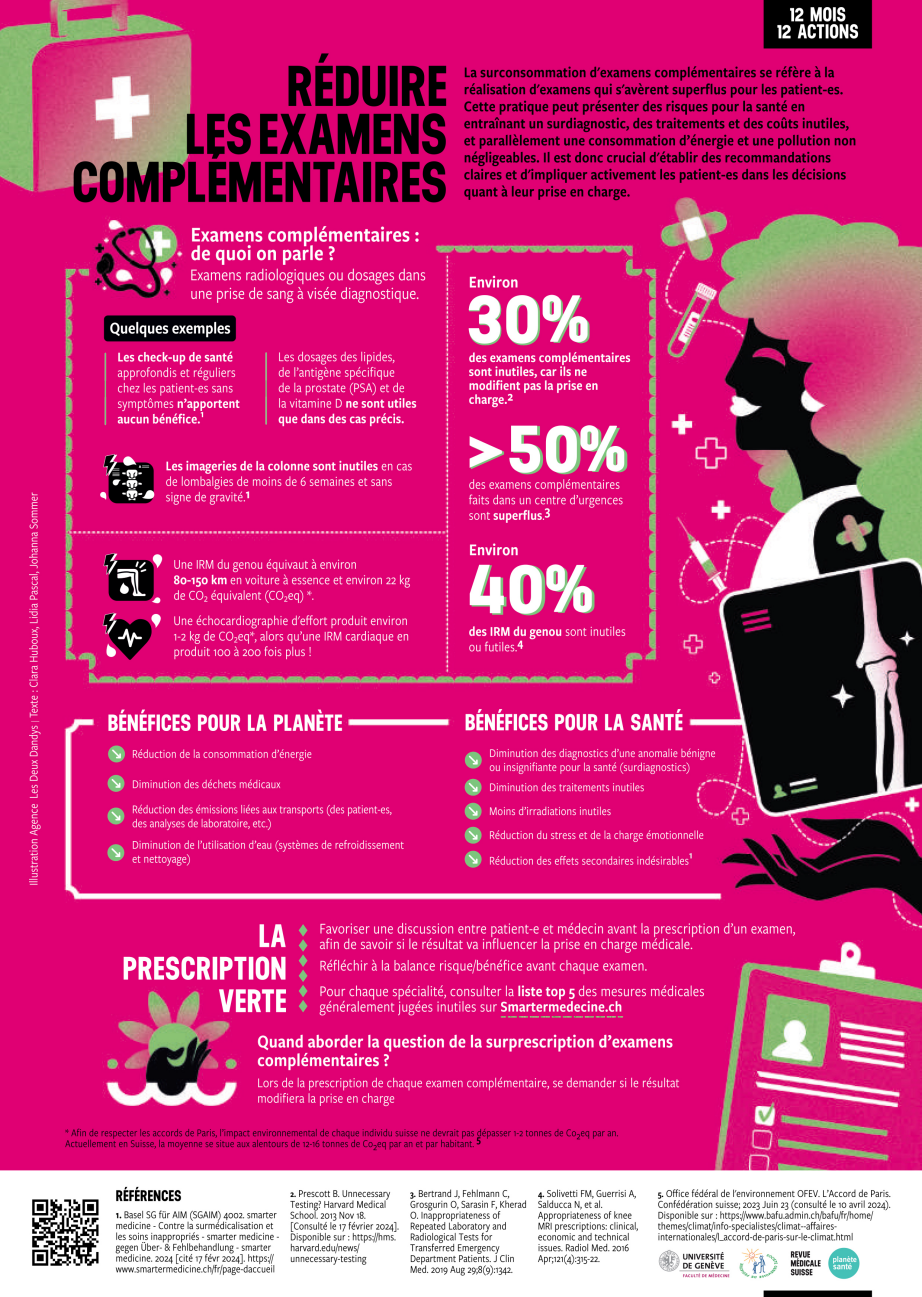
12 months/12 actions poster (in French). A collection of evidence-based “green prescriptions”, that are beneficial for both the individual health of patients and the environment. For more information, please consult [https://www.revmed.ch/infos-patients/] (retrieved 8^th^ January 2025)

During the next consultation, using role-modeling [11], you show your student how the May 2024 “12 months/12 actions” poster on “Reducing Additional Tests” (see figure 2 (Fig. 2)) can be included in a conversation with the patient, who comes with a first episode of shoulder pain and who had told the secretary that she needed the doctor “to write a prescription for a Magnetic Resonance Imaging (MRI) of her shoulder”.

You explain to your student an MRI is not appropriate in this situation and that after you have assessed the patient, you will explain what you recommend: rest from gym (you know that she works out 5 times a week) and physiotherapy plus analgesics on demand. You show Sabine the poster on the RMS website, which states that thirty percent of additional tests are “useless” and that studies show that forty percent of knee MRI’s, for example, don’t change management.

As a clinical teacher, you give pre-consultation instructions to Sabine to make sure she stays active in her observer role, i.e. to look out for the communication skills you use during the discussion with the patient. You ask Sabine to report afterwards on how you use the poster information to inform the patient on the benefits of reducing unnecessary tests for her health and for the environment.

The consultation goes according to plan and the patient is grateful to hear she doesn’t need an MRI after all. Sabine contributes a comment at the end of the interview by adding that she thanks the patient for helping to reduce unnecessary medical tests, thus avoiding waste of resources.

Feedback after the consultation reveals that Sabine noticed how you explained in a clear manner that the patient’s shoulder would heal with rest and physiotherapy plus analgesics on demand. She points out how a patient-centered approach with shared-decision making enabled the patient to avoid overdiagnosing with the co-benefit of reducing her carbon footprint.

Finally, Sabine reflects that she had not realized that family doctors, who have a long-term relationship with their patients, could make a significant impact on the use of medical resources and the environment in everyday discussions with their patients.

## Third “teachable moment” for “planetary health”: Smarter medicine

During the debriefing of a consultation with an older patient, Sabine refers to the smarter medicine guidance that advises against testing and treatment for dyslipidemia in the context of primary prevention in elderly patients. After discussing the medical background to this recommendation, you ask Sabine if she can think of any other positive aspects of the recommendation.

**Figure 3:**
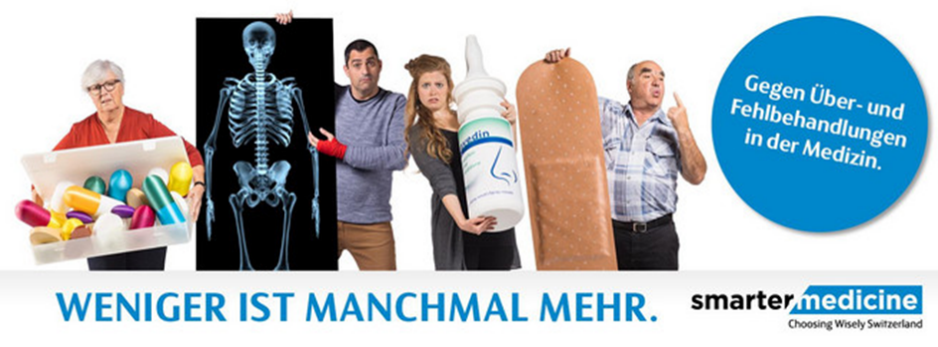
Campaign on the smarter-medicine website: [https://www.smartermedicine.ch/de/angebot/kampagne] (retrieved: 8^th^ January 2025)

The “smarter medicine” or “choosing wisely” campaigns are well-known initiatives that aim to promote optimal clinical care, emphasizing the importance of restraint in the use of medical tests and treatments (*“less is more”*). These campaigns seek to counter the overuse of medical tests and treatments that can cause harm to patients and offer no additional benefit [12]. Sabine correctly replies, that overutilization of health care is a main driver for health care costs and requires many resources [13]. Using the example of the older patient, you explain to Sabine what impact the recommendation can have on Planetary Health. The process of testing lipid status is associated with direct medical costs and the consumption of resources. In the event of inappropriate therapy, further costs are incurred, e.g. for follow up consultations, and medicines that need to be manufactured, packaged and transported, are consumed. In addition, the occurrence of adverse effects, such as muscle complaints, may necessitate further diagnostic procedures and therapeutic interventions, which in turn will have an economic and ecological footprint (see figure 3 (Fig. 3)).

Sabine is impressed by the idea of how many packs of medication this individual patient will accumulate, assuming the patient will statistically live for another 5-10 years. Especially when she considers how many packs would probably be used unnecessarily at national or international level. She quickly realizes that the recommendations of smarter medicine inevitably have a positive economic and ecological impact beyond the immediate clinical focus. The positive feedback from Sabine and the observation that she has developed a new perspective on the subject of medical care and Planetary Health motivates you to try to increase the awareness on this topic with all future students.

## Fourth “teachable moment” for “planetary health”: The Ecodoc project

Sabine is at the end of her 10 half-days immersion in family medicine practice. You invite her together with your team at the restaurant to share a meal and a pleasant moment. Sabine tells you that she really appreciated her time at your practice. She is convinced of becoming a general practitioner (GP). She tells you how she imagines the family practice of her dreams. She would like to work interprofessionally, with an approach focused on the needs of the patient. The practice would have to be well designed to offer a variety of services to patients. But what's most important to her is that the future practice should be as sustainable as possible.

**Figure 4:**
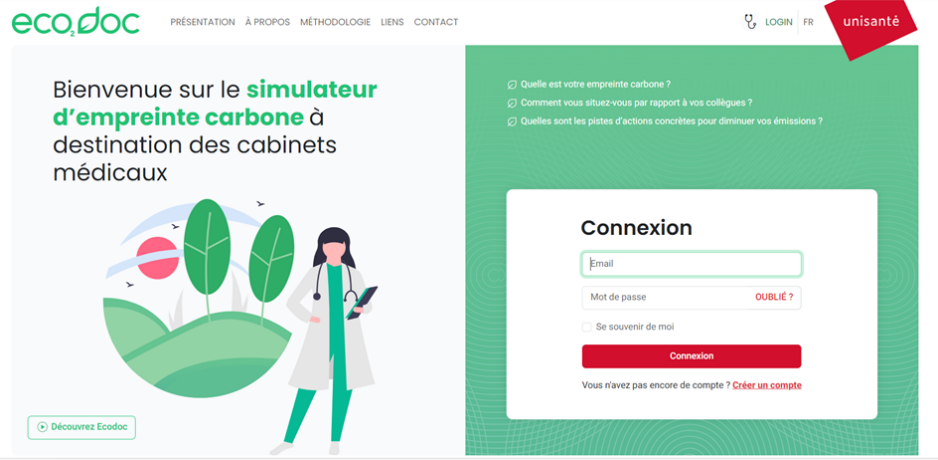
Ecodoc online carbon footprint simulator for family doctors: [https://www.eco-doc.ch/] (retrieved 8^th^ January 2025)

A few days ago, she spoke to a fellow student who is doing a placement with a family doctor involved in a study on the carbon impact of medical practices. That’s how she learned that there is an online carbon footprint simulator for family doctors that suggests concrete actions to limit the carbon impact of their practice [14]. You’ve never heard of it before, and you're looking forward to trying out this tool (see figure 4 (Fig. 4)).

An average family practice emits around 30 tonnes of CO_2_ equivalent per year. It has been shown that non-clinical activities such as staff and patients commute, energy use in buildings, waste management, etc., account for more than 70% of of the CO_2_ emissions associated with practices [15].

The Ecodoc project ([https://www.eco-doc.ch/], retrieved 8^th^ January 2025) has been developed to provide doctors with a tool that makes it easy to assess the carbon footprint of their practice. All they need to do is enter the general characteristics of the practice (number of staff, medical specialities, type of building, etc.), energy and hot water consumption, staff mobility, patient mobility, subcontracting and consumables.

Once all the data has been entered, the carbon footprint is estimated. The practice can compare its results with those of an average practice. A specific tab presents a list of possible actions, according to their potential to reduce carbon emissions in the specific context of each practice.

The following weekend, you decide to try out the carbon footprint simulator. You learn that you can reduce your carbon footprint by 8% just by cutting your heating by 1 degree. Several other actions seem easily achievable to your practice. You write an e-mail to Sabine to thank her for the tip and you look forward to presenting the tool to future students at the practice.

## Conclusions

In this article, we highlight some practical opportunities for teaching planetary health and sustainable healthcare from a family medicine perspective in undergraduate medical education.

The topic is still very young and to some extent conceptually immature in order to represent a defined curriculum within medical studies, which, according to the principle of constructive alignment, would require that the subject is not only talked about, but taught, the content implemented and the results evaluated as part of an assessment. The statements made by the British Medical Schools Council in 2022 regarding education for sustainable healthcare are also articulated in this respect, but nevertheless remain very general and essentially related to the pure knowledge aspect of medical education [16].

In a realistic setting, we have to face the facts that planetary health is neither a core competence for prospective doctors nor do most teaching GPs have expertise in sustainable healthcare. In addition, the programs for medical studies are already overcrowded. This means that for every hour of teaching in planetary health and sustainable healthcare, one hour of clinical teaching would have to be omitted, which is difficult to implement in the curriculum from both a planning and a student perspective. One option is to teach planetary health and sustainable healthcare as an elective part of the studies program, but this does not benefit all students. Another option is to offer the topic extra curriculum, but this is for various reasons not particularly popular. Our approach, therefore, is integrative.

We would like to encourage the integration of planetary health and sustainable healthcare within the family medicine curricula, which primarily take place in the clinical semesters. To this end, we show four possible ways in which this could be done in teaching practices, with the help of existing teaching materials and checklists. We believe that GP practices, which have clear ownership and management structures, due to their small size, are able to implement this economically sensible cross-cutting topic flexibly and without much additional effort on the part of the teaching physician. Furthermore, we also believe that students, with their youthful openness and curiosity, as well as their already existing knowledge in environmental protection and ecology, are able to critically examine and comment on the workflows and infrastructure of GP practices from the perspective of sustainability. We believe that this can lead to the initiation of a process of bidirectional learning that can result in a change in understanding but also in behavior for both the teaching practice and the student. This point of view is also emphasized in the Association for Medical Education in Europe (AMEE) consensus statement “planetary health and education for sustainable health care” [17].

On the one hand, this process can lead to improvement in sustainability in practice. On the other hand, it can represent a further factor that arouses the students’ interest in becoming a GP themselves, and thus taking the topic in question into their own hands in a self-determined way. But let’s be careful: a recently published study showed that wealthy people from wealthy countries tend to massively underestimate their personal carbon footprint [18]. The saying “do good and talk about it!” is, when applied to partial aspects of planetary health and sustainable healthcare, by no means sufficient for practicing medicine in a truly sustainable way. We must beware of virtue signaling!

With this in mind, it should be all the more motivating to remain aware of the planetary health and sustainable healthcare issues and to question our personal actions self-critically, as well as to look for and adopt concrete solutions that meet the needs of sustainability and the future of our profession, while guaranteeing patient safety. And finally, let us not forget, that poverty is one of the greatest risks to compromised health. Across the lifespan, residents of impoverished communities are at increased risk for mental illness, chronic disease, higher mortality, and lower life expectancy [19], [20]. These serious topics of environmental pollution and poverty are intertwined and have to be raised by us family physicians not only in our everyday work as doctors with the patients, but also as teachers with our students.

## Authors’ ORCIDs

- Telemachos Hatziisaak: [0000-0002-3745-4722]
- Olivier Pasche: [0000-0002-1202-9199]
- Andreas Plate: [0000-0001-6981-9389]
- Baptiste Pedrazzini: [0000-0003-3747-072X]
- Luca Gabutti: [0000-0002-1929-6759]
- Arabelle Rieder: [0009-0006-6254-1920]

## Acknowledgements

The authors would like to thank Dr. Moa Haller from the Bern Institute of General Practice for her contribution to the editorial process in developing the article.

## Competing interests

The authors declare that they have no competing interests.
